# Aging of mesenchymal stem cell in vitro

**DOI:** 10.1186/1471-2121-7-14

**Published:** 2006-03-10

**Authors:** Mandana Mohyeddin Bonab, Kamran Alimoghaddam, Fatemeh Talebian, Syed Hamid Ghaffari, Ardeshir Ghavamzadeh, Behrouz Nikbin

**Affiliations:** 1 Hematology-Oncology & BMT Research Center, Shariati Hospital, Tehran University of Medical Sciences, Iran; 2 Immunogenetics lab, Dept. of Immunology, College of Medicine, Tehran University of Medical Sciences, Tehran, Iran

## Abstract

**Background:**

A hot new topic in medical treatment is the use of mesenchymal stem cells (MSC) in therapy. The low frequency of this subpopulation of stem cells in bone marrow (BM) necessitates their in vitro expansion prior to clinical use. We evaluated the effect of long term culture on the senescence of these cells.

**Results:**

The mean long term culture was 118 days and the mean passage number was 9. The average number of PD decreased from 7.7 to 1.2 in the 10th passage. The mean telomere length decreased from 9.19 Kbp to 8.7 kbp in the 9th passage. Differentiation potential dropped from the 6th passage on. The culture's morphological abnormalities were typical of the Hayflick model of cellular aging.

**Conclusion:**

We believe that MSC enter senescence almost undetectably from the moment of in vitro culturing. Simultaneously these cells are losing their stem cell characteristics. Therefore, it is much better to consider them for cell and gene therapy early on.

## Background

Mesenchymal stem cells (MSC) are of great therapeutic potential because of their ability to self-renew and differentiate into multiple tissues[1]. They enhance engraftment of donor hematopoietic cells after co-transplantation in animal models[2]. In humans, MSC have been used to regenerate the marrow microenvironment after myeloablative therapy[3]. Possible clinical applications proposed for MSC include: stem cell transplantation[4], stem cell strategies for the repair of damaged organs[5] and gene therapy[6]. Friedenstein first described[7] MSC in bone marrow (BM) as a very rare population (0.01% to 0.001%), and Wexlel et al[8] reported a 1 in 3.4 × 104 frequency for these cells. We estimated that about 1 in 3.1 × 104 bone marrow mononuclear cells are MSC (unpublished data). Their level is even lower in cord blood9 and peripheral blood. Thus, it is essential to culture and populate MSC in vitro before putting them to therapeutic use. Scientific literature state that telomere length shortens after each division cycle. This leads to a gradual senescence. However, in vitro culture has proven that environmental conditions and differing treatment significantly increase or decrease culture life span. It seems as though depending on conditions cells are exposed to, rate of decrease in telomere length varies, leading to the observed differences. For example, Van Zglinicki et al reported that cells incubated in 40% oxygen soon stopped growing and, at the same time, had shortened telomeric DNA. They showed that telomeric DNA can be lost without cell division, perhaps by single-strand breaks in DNA[10].

With this in mind, we designed a study to evaluate the effect of long term invitro culture on the proliferation, differentiation, telomere length, phenotype, and morphology of these cells.

## Results

### The results of MSC culture

Three days after plating BM mononuclear cells in culture flasks, the mean number of fibroblast like cells were about 6800 (90 cell/cm2) of total heterogeneous cells. Gradually, the fibroblast like cells (MSC) increased and the heterogeneous cells decreased in the culture.

**Figure 1:**
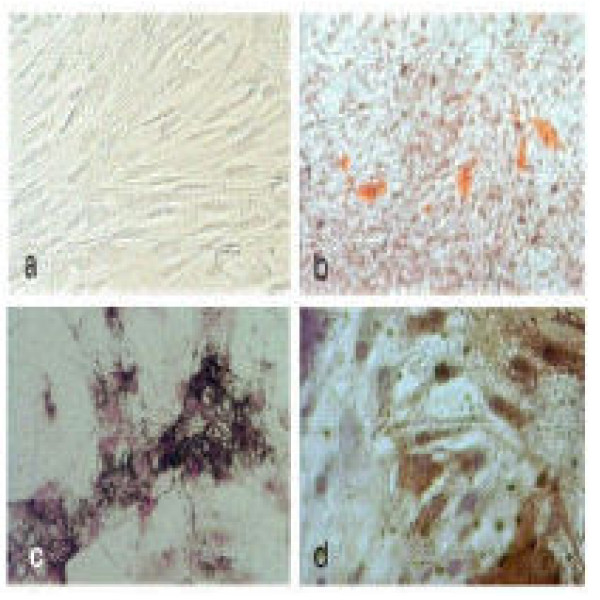
a) Bone Marrow MSC in conventional culture (adherent, elongated cells); b) Oil red O staining showing fat accumulation in adipocytes. c & d) Van Kossas and Alkaline Phosphatase staining showing osteocytes differentiation of MSC.

The predominant cell after 2 weeks of culture was fibroblast like (Fig. 1a) and dislodged readily on trypsinization in 3 minutes.

Primary culture took between 11 and 25 days (mean: 15.6 day). The total count of harvested cells from primary culture was between 5.4 × 105 and 39.0 × 105 (mean: 17.0 × 105 cell). The mean number of harvested MSC and the mean time of confluency from the second culture on is shown in table 1. As can be seen in table 1, the average number of cells that proliferate during each passage and the average confluency time in the various passages are different. To analyze these differences we performed the Repeated Measurement Analysis of Variance statistical test, but we did not find any significant difference between the different passages.

**Table 1:** The result of the mean and SD of BM-MSC culture.

passage number	Mean number of harvested cell (× 10^4^) ± SD	Mean time of counfluency (day) ± SD	Mean number of PD ± SD	Mean number of telomere length (Kbp) ± SD
1^th ^passage	170 ± 105.26	15 ± 4.23	7.7 ± .55	9.2 ± .60
2^th ^passage	404 ± 190.72	10 ± 4.78	3.1 ± .67	-
31^th ^passage	280 ± 148.52	12 ± 4.10	2.6 ± .68	9.0 ± .56
4^th ^passage	283 ± 103.55	12 ± 1.75	2.6 ± .58	-
5^th ^passage	258 ± 109.73	14 ± 4.76	2.5 ± .56	8.9 ± .58
6^th ^passage	232 ± 122.26	16 ± 5.94	2.3 ± .61	-
7^th ^passage	206 ± 152.95	14 ± 5.27	2.0 ± .81	8.8 ± .56
8^th ^passage	175 ± 77.57	14 ± 4.71	1.9 ± .69	-
9^th ^passage	210 ± 134.06	17 ± 4.65	2.1 ± .82	8.7 ± .75
10^th ^passage	109 ± 61.68	15 ± 3.35	1.2 ± .73	-

To evaluate whether donor age affects in vitro cell senescence, we divided our donors into two groups. The first group included 6 individuals under the age of 10, and the remaining 5 who were between the ages of 23–63 made up the second group.

Independent sample Test and Repeated Measurement Analysis was performed to evaluate telomere length, PD level, harvested cell dose and confluency time for each passage. No significant difference was observed.

The goal of this study was keeping the MSC in the culture by multiple passages up to 120 days. Nine of the 11 samples survived between 100 and 164 days and were passaged between 8 to 10 times. Two samples survived up to 63 and 95 days, and were passaged 5 and 6 times respectively (they didn't undergo the full procedure due to contamination).

### Immunophenotype of MSC

The mean percent of CD expression of the three antigens in 5 different passages was compared using Repeated Measurement Analysis of Variance test. Results demonstrate no significant difference in their expression. These cells stained positively for CD44 and CD13 but negative or very low positive for CD34.

### Differentiation of BM MSC

MSC were differentiated along adipogenic and osteogenic lineages. Oil red O staining for adipocyte and Van Kossas and Alkaline phosphatase staining for osteocyte were positive (Fig. 1b). Adipogenic differentiation of MSC after 2–3 weeks incubation in adipocyte culture media demonstrated scattered aggregates of oil red o positive staining cells. But within the same time limit, the osteocyte culture media exhibited a homogeneous and relatively abundant number of Van Kossas positive cells.

The differentiation ability of MSC varied in the 5 different passages. Every one of the samples cultured in osteogenic and adipogenic mediums differentiated to osteocyte and adipocytes by the 4th culture. In the 8th passage, 25% (2 of 8), and in the 10th passage, 20% (1 of 5) of the samples lost their osteogenic differentiation potential. For adipocytes, in the 6th passage 10% (1 of 10), in the 8th passage 50% (4 of 8), and in the 10th passage 60% (3 of 5) of the samples lost their adipogenic differentiation potential. The percentage of samples with the ability to differentiate into osteocyte and adipocyte in advancing CPD is shown in table 2.

**Table 2:** The percent of samples which differentiate to adipocyte and osteocyte in ongoing CPDs after 2–3 week culture period is shown here.

CPD No.	Adipocytes	Osteocytes
10	100 %	100 %
15	100 %	100 %
20	90 %	90 %
25	50 %	75 %
30	40 %	80 %

### Long-term growth kinetics

To examine long-term growth kinetics of MSC culture, we measured cumulative population doublings (PDs), with respect to the passage number in multiple donors. The average number of cumulative PD in 130 days was about 30 (range 26–32). MSC underwent an average of 7.7 PDs prior to the first passage. The mean PD and growth kinetics of different samples upon subsequent passages is shown in table 1 and Figure 1d, respectively.

### Morphological characteristics of BM MSC in long-term culture

After a long period of normal growth, MSC culture showed abnormalities typical of the Hayflick model of cellular aging in cultured human fibroblasts 12. The cells varied in size and shape, the cytoplasm began to be granular with many cell inclusions, and debris was formed in the medium. On average, granules were noted in the cytoplasm after 84 days (range: 62–122), while debris formation was observed in medium after about 72 days (range: 35–97) after primary culture. However, in later stages, around day 120 in some cultures, the granular cell began to be vacuolated, rounded and finally detached from the base of the flasks.

### Telomere length of MSC

**Figure 2:**
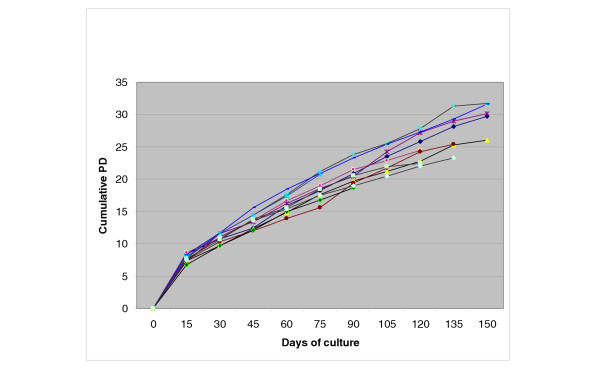
Long term growth curves; each obtained from an individual donor.

The range of the mTRFL varied between 10.2 kbp to 7.8 kbp in different BM samples and in different passages. mTRFL decreased from an average of 9.19 kbp in the first passage to 8.75 kbp in the 9th passage (Fig. 2), giving a telomere shortening of about 0.44 kbp within 120 days of expansion (table 1).

## Discussion

Since MSC can differentiate into multiple tissues in vitro and in vivo, they attract a lot of attention in cell and gene therapy. The low frequency of MSC in BM[7,8] necessitates their in vitro expansion prior to clinical use. It is known that when the cells become senescent, they are unable to proliferate further. As a result, it is necessary to evaluate the proliferative capacity of expanded MSC to maintain long-term tissue regeneration before re-infusion.

The key feature of senescence in dividing cells is a long period of normal growth followed by cessation of growth. So, throughout their life span, certain events must bring about senescence. Our study has shown a number of changes in physiological, functional, and molecular parameters that occurred during long-term cultures. These changes included:

- Typical Hayflick Phenomenon of cellular aging

- Gradual decreasing proliferation potential

- Telomere shortening

- Impairment of functions

The actual age of a culture is normally recorded in population doublings (PDs). Colter et al[13] reported that the single-cell-derived colonies of MSC can be expanded up to as many as 50 PDs in about 10 weeks. But we found that MSC can be expanded up to 30 PDs, similar to Kassem et al11, in about 130 days (18 weeks).

The curve relationship between cumulative PD and duration of culture demonstrates a relatively linear decreasing PD rate with the progression of time. Furthermore, an appreciable decrease in the number of PD was seen in the latter days (more than 130 days in culture), suggesting that MSC proliferative potential decreases faster after 120 days in vitro expansion. To investigate the aging of MSC morphologically, we studied the culture for the presence of any abnormalities in cell morphology. Production of debris in the medium and granules in the cytoplasm has been used as markers for fibroblast aging in vitro[14]. In the present study, granulated cytoplasm and debris was observed and increased from the third month on.

We investigated another marker for cellular senescence, normally the mean telomere length. In the absence of the enzyme telomerase, telomeres gradually get shorter as cell division proceeds, and shortening of telomere throughout the life span is well-documented[15]. The consequence maybe the inactivation of genes closest to the telomere sequences, either directly or indirectly by a position effect, perhaps involving the formation of heterochromatin. However, maintenance of telomere length has been observed in germ cells, some stem cells and malignant cells due to activation of the telomerase enzyme[16].

Our present study shows that MSC senescence is associated with telomere shortening during in vitro expansion. The rate of telomere shortening was 100 bp in every two passage. Other studies[17,18] have shown that the telomere length of MSC shortens in culture expansion. However the rate of telomere shortening in Kassem et al[11] study was 100 bp/PD, which is very high in comparison to our study. This difference maybe due to the long period of their culture (> 500 days) in comparison to our study (120 days). They showed that mTRFL decreased from an average of 10.4 kb in early passage cells to 7.1 kbp in late passage cells. Although loss of mTRFL in later passages is high but the mean amount of shortening was divided by the cumulative PD number. This could partly explain the difference.

In addition to the telomere length shortening, we investigated the osteogenic and adipogenic differentiation capacity of MSC. MSC differentiation into other lineages has been used as a marker for the multipotential nature of these cells[19]. In our study, the differentiation potential to adipocyte and osteocyte dropped in the late-passages, similar to previous reports[20,21]. Our study also demonstrated that, in the later passages, a greater number of samples lose their adipocyte differentiation potential in comparison to osteocytes in the same condition. At the level of one culture plate, the percentage of cells that differentiated into adipocyte were lower than those differentiating into osteocytes.

It is generally agreed that senescent cells are irreversibly blocked in cell division, but they are still capable of many other cell functions. According to the telomere theory of cellular senescence, the shortening of chromosome ends would itself trigger a cell cycle block.

Stenderup et al.[11] showed that donor age affects rate of in vitro senescence in MSC. They define their old age group as above 66 years of age, and compare this with a group of donors ranging from 18–29 years. The difference they observe is significant. In our study, no significance in age difference was observed. This could be directly due to the donor age we had (all below 63 and six below 10).

When there is talk of aging, transformation and tumorigenesis inevitably lingers in the background. In some cases, we observed changes from elongated adherence cells to rounded non-adherent cells after 120 days. Today, this phenomenon is noted as a mark of transformation into cancerous cells. Interestingly, recent studies highlight this issue in invitro culturing. Rubiro et al.[22] demonstrated that adipocyte tissue derived hMSC under long term culture conditions, upon entering old age, spontaneously transform into small, clustered aggregations displaying molecular and chromosomal changes and abnormalities at a rate of 50% or so. Serakinci et al.[23] report a similar change in long term culture. They note that transduced cells develop characteristics which correlate with the acquisition of tumorigenic phenotype (loss of contact inhibition, cell cycle regulation alteration, senescence, ...) in PDL above 70. They report that these cells do not undergo such alterations in lower PDs. It is notable that as cells age, transformations become more common.

## Conclusion

Our results suggest that MSC enter senescence and start to lose their stem cell characteristics almost undetectably from the moment in vitro culture begins. MSC should therefore be considered for cell and gene therapy only at the early stages of in vitro culture.

## Methods

### Sample collection and MSC cultures

Ten ml of BM was obtained from 11 (5 female, 6 male) healthy hematopoietic stem cell transplant donors. None of the donors had used any medication or growth factor at the time of collection. Informed consent according to an approved protocol by the internal medicine ethics committee was obtained. The mean age of BM donors was 25 years (range: 2.5 to 63).

The mononuclear cells (MNCs) were isolated from the collected samples by the Ficoll density gradient method. Then, they were washed in RPMI 1640 and resuspended at 1 × 106 cells/ml in culture medium [Dulbecco's modified Eagle medium, low glucose (DMEM-LG), 10% (v/v) heat-inactivated fetal bovine serum (FBS) and penicillin/streptomycin (all from Gibco)]. Twenty-one ml of Cell suspension was plated in a 75 cm2 flask (Greiner bio-one) for primary culture.

Flasks were incubated at 37°C in a humidified atmosphere of 5% CO2 and fed by complete medium replacement every 4 days. When fibroblast-like cells at the base of the flask reached more than 90% confluence, adherent cells were detached using 0.25% trypsin EDTA and replated (passaged) at a density of 1 × 104 cells/ml in two 75 cm2 flasks. On reaching confluence, all cultures were passaged sequentially up to 120 days. For every even passage, some of the expanded MSC was separated for adipocyte and osteocyte differentiation potential and immunophenotype testing. For every odd passage, the cells were examined for telomere length.

### Immunophenotyping

Surface expression of CD44, CD13 and CD34 was determined on culture-expanded MSC. The monoclonal antibodies used were anti-CD44 fluorescein isothiocyanate (FITC), anti-CD13 phycoerythrin (PE), and anti-CD34 FITC (all from Dako). Relevant isotope control antibodies were also used. Flow cytometry was performed on a FACScan (Becton Dickinson), and data were analyzed with Cellquest software.

### Differentiation of MSC

Adipogenic differentiation was assessed by incubating the cells with DMEM-LG and 10% FBS supplemented with 0.5 μM hydrocortisone, 0.5 μM isoboutyl methylxanthine, 60 μM indomethacin (Sigma-Aldrich), and 10 μg/ml insulin (R&D) for 2–3 weeks. Adipocyte is recognized by the accumulation of lipid-containing vacuoles which stain red with Oil red O.

Osteogenic differentiation was assessed by incubating the cells with DMEM-LG and 10% FBS supplemented with 0.1 μM dexamethasone, 10 μM β-glycero-phosphate, and 50 μM ascorbate (all from Sigma-Aldrich) for 2–3 weeks. To assess mineralization, cultures were stained with silver nitrate (Von Kossa's staining).

### Determination of population doubling level and morphological characteristics of long term culture

Long-term cell growth in vitro is a sensitive method to detect subtle changes in the kinetics of proliferation of the cell population. To determine the number of cumulative population doublings, BM mononuclear cells were seeded 1 × 106 cell/ml (28 × 104 cell/cm2) in T-75 flasks. The adherent MSC were counted after 3–4 days to quantify the initial number. At confluence, the cells were trypsinized, counted and reseeded at a density of 1 × 104/ml (2.8 × 103 cell/cm2). The numbers of PDs were calculated using the formula logN/log2 11, where N is the cell number of the confluent monolayer divided by the initial number of cells seeded. This procedure was repeated in every passage.

To study the morphological characteristics, cell culture flasks were observed every time at medium re-feeding intervals (every 4 day), to detect any abnormalities in cell morphology and medium. When any variation was observed, the changes were recorded in the culture files.

### Determination of telomere length

For telomere length assay, we used the "Telo TAGGG Telomere Length Assay" kit (Roche Molecular Biochemical) according to the manufacturer's recommendations. Briefly, the test principle is as follows: The genomic DNA was isolated and digested using restriction enzymes *RsaI *and *HinfI *for 2 h at 37°C. The DNA fragments were separated by gel electrophoresis for 2–4 h at 70 V on a 0.8% agarose gel and transferred to a nylon membrane by Southern blotting.

The blotted DNA fragments are hybridized to a digoxigenin (DIG)-labeled probe specific for telomeric repeats and incubated with a DIG-specific antibody covalently coupled to alkaline phosphate. Finally, the immobilized telomere probe is visualized by virtue of alkaline phosphatase metabolizing CDP-*Star*, a highly sensitive chemiluminescence substrate. The average TRF length can be determined by comparing the signals relative to a molecular weight standard.

After exposure of the blot to an X-ray film, the mean TRF length were scanned and calculated by the multianalizor (Bio-rad) software.

### Statistical analysis

All data are presented as mean ± SD. The data collected in ten passages was compared by Repeated Measurement analysis of variance test. P. values of less than P < 0.05 were considered significant.

## Authors' contributions

MMB: Cell culture, differentiation, Imuunophenotyping, Telomere Assay, and was the main designer and coordinator of the study

KA: Sample Aspiration

FT: participated in Cell culture and writing of the manuscript

SHG: participated in Telomere assay

AG: participated in coordination of study

BN: Supervisor and thesis advisor, participation in design of study

All authors read and approved the final manuscript.

**Figure 3:**
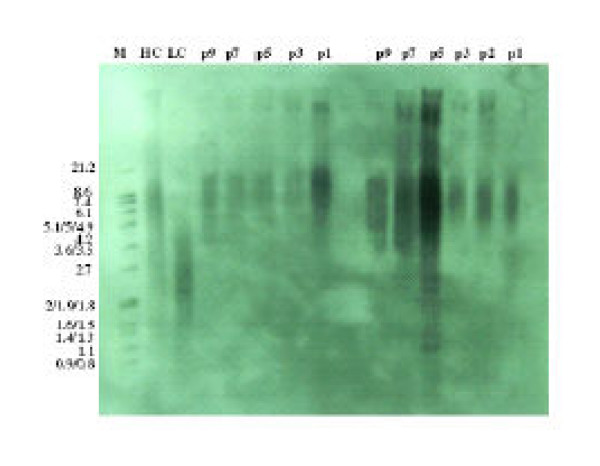
Southern-blot analysis of telomere lengths of expanded MSCs derived from two BM samples during multiple passages (CH = Control-DNA, High, CL = Control-DNA, Low, P = Passage).
